# Retraction: HLA-B35 and dsRNA Induce Endothelin-1 via Activation of ATF4 in Human Microvascular Endothelial Cells

**DOI:** 10.1371/journal.pone.0241404

**Published:** 2020-10-22

**Authors:** The PLOS ONE Editors

After this article [1] was published, concerns were raised about results reported in Figs 3 and 4. Boston University investigated this work and determined that images in Figs 3 and 4 had been inappropriately duplicated, manipulated, and mislabeled.

Specifically:

- Fig 3A (left) Lamin A/C lanes 2–4 and Fig 3C (left) Lamin A/C panel appear to report the same data with aspect ratio adjusted.
- Fig 3B (left) ATF6 lanes 3–5, Fig 3C (left) ATF6 panel, and Fig 3C (right) eIF2α panel appear to report the same data with aspect ratio adjusted.
- Fig 3B (left) Lamin A/C lanes 1–4 and Fig 4D Lamin A/C panel appear to report the same data.
- Fig 3A (right) eIF2α and Fig 3B (right) PERK appear to report the same data.
- The Fig 3C β-actin panel and Fig 4C eIF2α panel were mislabeled and report data that do not correspond to the experiments and/or sample labels described in the figures and their legends.

The authors requested retraction of the article and stated that the underlying data for these results are not available.

In light of the above concerns, and in line with the institution’s recommendation, the *PLOS ONE* Editors retract this article.

SLe, GAF, FRP, SLa, RL, and MT agreed with the retraction, though SLe noted that she stands by the article’s findings. SLe, GAF, SLa, and MT apologize for the issues with the published article. IC and RS either could not be reached or did not respond directly.
